# miRNAs in decidual NK cells: regulators worthy of attention during pregnancy

**DOI:** 10.1186/s12958-021-00812-2

**Published:** 2021-10-02

**Authors:** Liman Li, Ting Feng, Weijie Zhou, Yuan Liu, Hong Li

**Affiliations:** Center of Translational Medicine, Key Laboratory of Birth Defects and Related Diseases of Women and Children of Ministry of Education, West China Second University Hospital, Sichuan University, Chengdu, China

**Keywords:** Decidual NK cells, miRNAs, Immune regulation, Pregnancy

## Abstract

The critical immune effectors, including T, B, and natural killer (NK) cells, dendritic cells, and macrophages participate in regulating immune responses during pregnancy. Among these immune cells, decidual NK (dNK) cells are involved in key placental development processes at the maternal–fetal interface, such as uterine spiral artery remodeling, trophoblast invasion, and decidualization. Mechanistically, dNK cells significantly influence pregnancy outcome by secreting cytokines, chemokines, and angiogenic mediators and by their interactions with trophoblasts and other decidual cells. MicroRNAs (miRNAs) are small non-coding RNA molecules that participate in the initiation and progression of human diseases. Although the functions of circulating miRNAs in pathological mechanism has been extensively studied, the regulatory roles of miRNAs in NK cells, especially in dNK cells, have been rarely reported. In this review, we analyze the effects of miRNA regulations of dNK cell functions on the immune system during gestation. We discuss aberrant expressions of certain miRNAs in dNK cells that may lead to pathological consequences, such as recurrent pregnancy loss (RPL). Interestingly, miRNA expression patterns are also different between dNK cells and peripheral NK (pNK) cells, and pNK cells in the first- and third‐trimester of gestation. The dysregulation of miRNA plays a pivotal regulatory role in driving immune functions of dNK and pNK cells. Further understanding of the molecular mechanisms of miRNAs in dNK cells may provide new insights into the development of therapeutics to prevent pregnancy failure.

## Introduction

A delicate immune balance is crucial for maintaining pregnancy, and disruption of this balance can lead to certain pregnancy complications, such as recurrent pregnancy loss (RPL), pre-eclampsia (PE), and fetal growth retardation (FGR) [1]. The maternal–fetal interface consists of various immune cells, decidual stromal cells, and trophoblast cells. These fetal and maternal cells can contact each other to maintain immunologic tolerance and normal fetal growth [2]. The presence of innate and adaptive immune cells is one of the characteristics of early pregnancy [3, 4], including approximately 70 % of decidual NK (dNK) cells [5], 20–30 % of macrophages [6], 1.7 % of dendritic cells [7], and 3–10 % of T cells (including Treg cells and γδT cells) [8]. Advances in understanding the mechanisms by which NK cells modulate the immune system during pregnancy have increased in recent years [9–12]. Evidence also supports that microRNAs (miRNAs) are involved in immune cell development, which may be selectively regulating the placental development as well as subsequent conceptus viability [13]. In this review, we highlight the roles of dNK cells in pregnancy and the complicated interplays with miRNA regulatory networks. A better understanding of the miRNA regulation of NK cells in pregnant women and their functional specificity could open a new avenue for translational applications in precision medicine and miRNA-based therapeutic tools to protect the fetus and the mother.

## dNK cells in pregnancy

**Fig. 1:**
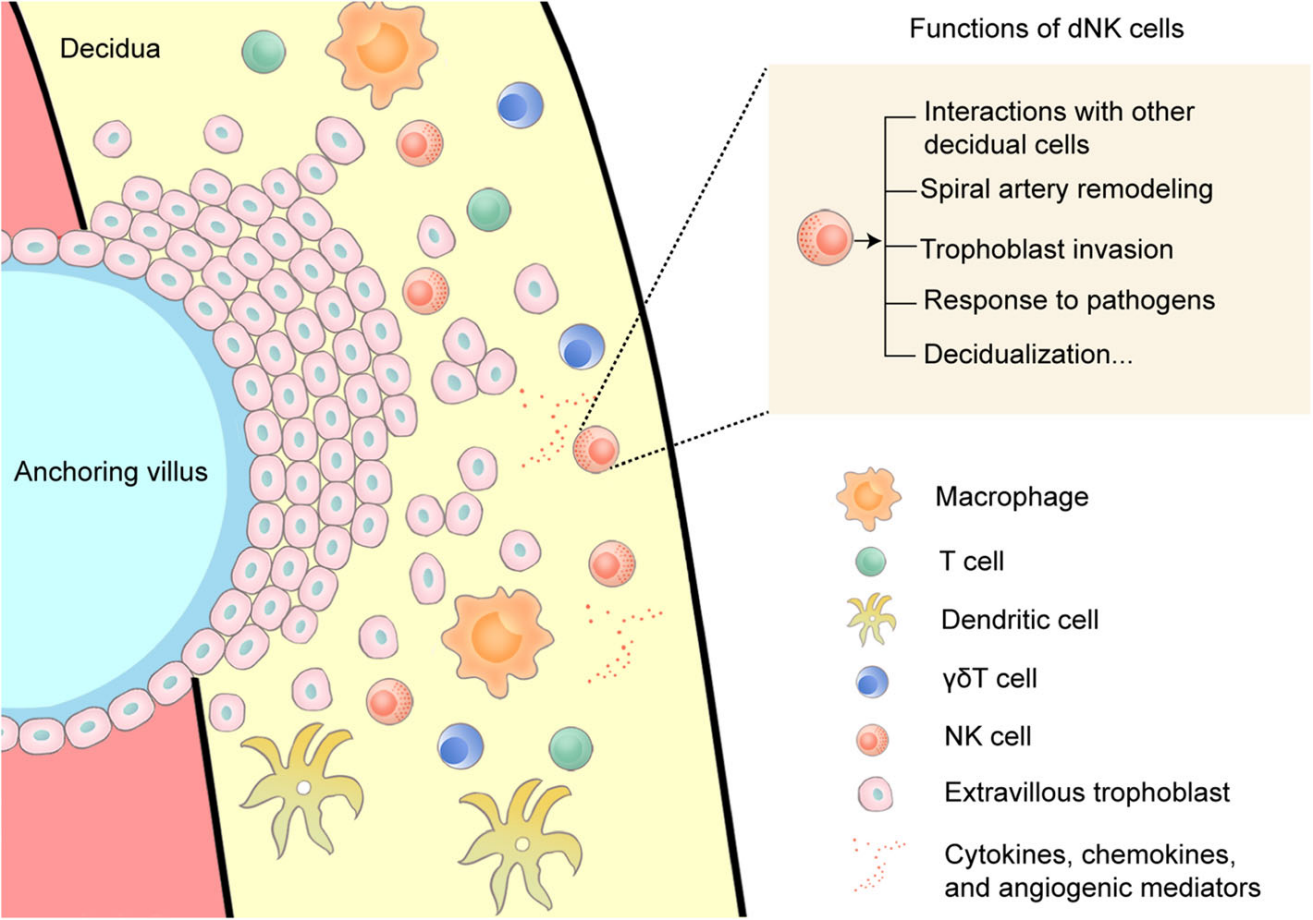
Diagram illustrating the immune cells at maternal–fetal interface. A large number of immune cells, including macrophages, T cells, dendritic cells, γδT cells, NK cells are present in decidua. They have capabilities of secreting cytokines, chemokines, and angiogenic mediators, and establishing interactions with other decidual cells. These functional immune cells are essential for maintaining the immune balance at maternal-fetal interface. Among them, decidual NK cells make remarkable contributions to spiral artery remodeling, trophoblast invasion processes, prevention of pathogenic invasion of fetal placenta, as well as decidualization, which play indispensable roles in pregnancy success. dNK cell, Decidual NK cell

A large number of immune cells, including macrophages, T cells, dendritic cells, γδT cells, NK cells are present in decidua during early pregnancy, and the three major leukocyte subsets are NK cells, cluster of differentiation (CD) 14 + myelomonocytic cells, and T cells [14]. Among them, dNK cells make remarkable contributions to spiral artery remodeling, trophoblast invasion processes, prevention of pathogenic invasion of fetal placenta, as well as decidualization, which play critical roles in pregnancy success **(**Fig. 1**)**. Decidual NK cells are a unique tissue-resident subpopulation and the most abundant cell type (accounting for around 70 % of decidual leucocytes) in first trimester of gestation [15]. Several studies have proposed that dNK cells originate from the migration of pNK cells or the differentiation of hematopoietic precursors CD34 + stem cells [16, 17]. Decidual NK cells are characterized by the CD56^bright^CD16^−^ surface phenotype with considerable immune regulatory cytokine production and high expressions of killer cell Ig-like receptors (KIRs) that are distinct from pNK cells and other tissue-resident NK subsets [18]. There is evidence for dNK cells’ abilities to produce a variety of cytokines, chemokines, and angiogenic mediators such as interferon (IFN)-γ, tumor necrosis factor (TNF)-α, interleukin (IL)-8, interferon gamma-inducible protein (IP)-10, and vascular endothelial growth factor (VEGF) that participate in immunomodulation during pregnancy [19, 20].

### Interactions of dNK cells/other decidual cells

The interactions between dNK and other decidual cells play key roles in maintaining maternal–fetal immune tolerance. For instance, CXCL16 derived from trophoblasts leads to M2 macrophage polarization that in turn modulates the killing ability of dNK cells [21]. Decidual NK cells are closely connected with decidual myelomonocytic CD14+ (dCD14+) cells. Following interaction with dNK cells, dCD14 + cells are capable of inducing regulatory T cells (Treg) cells. Mechanistically, IFN-γ secreted from dNK cells induces indoleamine 2,3-dioxygenase (IDO) up-regulation in CD14 + cells. In turn, IDO can induce the Treg cells by generating the tryptophan catabolite L-kinurenine. Interestingly, only the interaction between dNK and dCD14 + cells lead to Treg cell induction, whereas other coculture combinations such as NK and CD14 + cells isolated from peripheral blood are ineffective [22].

### dNK cells control spiral artery remodeling

Uterine spiral artery remodeling is one of the most important physiological changes in pregnancy. Pregnancy complications such as PE, FGR, and RPL are linked with impaired spiral artery remodeling [23, 24]. Successful uterine spiral artery remodeling depends on a series of sequential steps, including vascular dilatation, endothelial cell swelling, destruction of the vascular smooth muscle cell (VSMC) layer, and subsequent fibrinoid matrix deposition and invasion of extra-villous cytotrophoblasts. It has been shown that this process is regulated by the massive infiltration of maternal immune cells in the decidua [25, 26]. Decidual NK cells can secrete IL-8, IFN-γ, and TNF-α to initiate the destabilization of vascular structures and spiral artery transformation before extra-villous trophoblast (EVT) interaction [27, 28]. Decidual NK cell-derived IFN-γ could induce the up-regulation of lncMEG3 expression in VSMCs. Decidual NK cell/IFN-γ treatment and lncMEG3 overexpression inhibit VSMC proliferation and stimulate VSMC migration and apoptosis [29]. Furthermore, dNK cells also affect VSMC biology by secreting angiopoietin (ANG)-1and ANG-2, which promote VSMC to migrate into the stroma surrounding remodeling spiral arteries where they undergo apoptosis and are phagocytosed by resident macrophages [24].

### dNK cells regulate trophoblast invasion

Balanced EVT invasion regulation is key to gestation success [30]. Deficiency of invaded trophoblastic cells is the one of main causes of dysfunctional placentas. Inadequate motility of trophoblastic cells leads to abnormal remodeling of spiral arteries and disrupts material exchange between fetus and mother, thus causing a series of pregnancy-related disorders [31, 32]. Earlier study has revealed that trophoblast migration and invasion are correlated with the enhanced ability of dNK cells to produce IL-8 and IP-10, which bind to C-X-C motif chemokine receptor 1(CXCR1) and CXCR3 expressed by invasive EVT, respectively [33]. Additionally, dNK cells could promote trophoblast invasion by producing hepatocyte growth factor (HGF) and inducing trophoblast differentiation toward the endothelial phenotype by the secretion of VEGF-C and HGF [34]. In PE pregnancies, dNK cells exhibit a reduced promotion effect on first trimester trophoblast invasion and migration when compared to normal controls. In small-for-gestational-age (SGA) pregnancies, dNK cells show impairments in proliferation, migration, invasion, and tube formation of trophoblast cells or vascular endothelial cells compared to those in appropriate-for-gestational-age (AGA) pregnancies [35].

### dNK cells response to pathogens

Threatening pathogens can jeopardize the function of the maternal–fetal interface and spread to the fetus, with negative consequences. For instance, infections by TORCH pathogens can result in fetal growth retardation or death, organ injury, or other sequelae [5, 36]. Remarkably, dNK cells are able to infiltrate human cytomegalovirus (HCMV)-infected tissue and co-localize with infected cells. dNK cells become cytotoxic effectors when they are exposed to HCMV-infected cells and the killer cell lectin-like receptor K1 (also known as NKG2D), CD94/NKG2C, or 2E activating receptors participate in the acquired cytotoxic function. Activation of dNK cells could increase their abilities to respond to placental HCMV infection and limit the subsequent virus-induced placental pathology [37]. In addition, dNK cells can release IFN-γ to limit HIV replication in decidual macrophages and directly transfer granulysin (GNLY) to fetal cells by nanotubes, killing intracellular *Listeria monocytogenes* without killing the host cell [38, 39]. Taken together, dNK cells may reduce bacterial loads at the maternal–fetal interface as a result of their cytotoxic functions. More importantly, dNK cells can provide the immune defense that protects the target cells and is compatible with maintaining maternal–fetal immune tolerance. However, as new viruses emerge, such as coronavirus disease 2019 (COVID-19), which is associated with a high incidence of severe maternal and neonatal complications [40], further studies are urgently needed for a better understanding of the roles of dNK cells in protecting the mother and the fetus against new bacterial, fungal, and parasitic pathogens.

### dNK cells contribute to decidualization

Decidualization is necessary for normal implantation of the blastocyst [41]. Previous studies have exhibited that the absolute number as well as the percentage of uterine NK (uNK) in uterine immune cells are significantly decreased in endometrium of estrous mice, compared to pregnant mice. This result indicates that decidualization is accompanied by NK cell enrichment [42]. Furthermore, autocrine/paracrine factors are linked to endometrial decidualization, like parathyroid hormone-like hormone (PTHLH), cytokines, IL-11, and activin A [43, 44]. As for dNK cells, with the transformation of endometrial stromal cells (ESCs) into decidual stromal cells (DSCs) during decidualization, IL-24 derived from DSCs can induce CD56^dim^ uNK to differentiate into CD56^bright^CD16^−^ dNK with high immunomodulation and angiogenic activities, and low cytotoxic activity that benefits normal pregnancy [45]. Interestingly, dNK cells can secrete IL‑25 facilitating the decidualization of ESCs [46], which have a positive feedback effect on maternal-fetal interface.

## miRNA basics

miRNAs are a class of ∼22 nt non-coding RNAs that negatively regulate gene expression through directly binding the 3′UTR of mRNA targets [47]. The miRNA class is found in all eukaryotic cells and some viruses. Research in the past few years reported that miRNAs have potential as biomarkers for the early detection and therapeutic targeting of diseases as well as acting as important regulatory elements in healthy and cancerous tissues [48, 49]. Of note, miRNAs can be packaged within extracellular vesicles including exosomes and microvesicles, and these vesicles contain numerous miRNAs that transfer between cells, thereby establishing intercellular communication [50].

## Differentially Expressed miRNAs (DEMs) in dNK and pNK cells during pregnancy

### DEMs in pNK cells derived from different pregnancy time points

A previous study [51] performed real-time PCR-based arrays to explore the levels of 756 miRNAs in pNK cells at different time points (first, second, and third trimester and post-delivery) in pregnancy. The researchers provided a comparison of miRNA expression profiles and demonstrated a total of 25 miRNAs are significantly up-regulated and only 1 miRNA (miR‐7‐2‐3p) is significantly down-regulated in pNK cells in the third trimester compared to the first trimester. Among these dysregulated miRNAs, miR‐517a‐3p and miR‐518b, two placenta-derived miRNAs [chromosome 19 miRNA cluster (C19MC) miRNAs] are significantly up-regulated in the third trimester and down-regulated at 4 days following delivery, suggesting that the transfer of placental miRNAs into maternal pNK cells might occur during pregnancy. Researchers hypothesized these interactions are possibly mediated via exosomes [52].

### DEMs in dNK cells and matched pNK cells

Furthermore, another study [53] compared the miRNA expression pattern in dNK cells and their matched pNK cells. Results show there are 86 miRNAs significantly expressed in dNK and pNK cells samples. There are 48 miRNAs commonly expressed in both dNK and pNK cells but only 5 miRNAs expressed in dNK and pNK cells at similar levels. A great majority of miRNAs display differential expressions in the two NK cell populations. Of note, there are 28 up-regulated and 15 down-regulated miRNAs in dNK cells compared to matched pNK cells, and 36 exclusively expressed miRNAs in dNK cells whereas there are only 2 exclusively expressed miRNAs in pNK cells.

Validation analysis via qPCR revealed that miR-214 and miR-10b are expressed only in dNK cells, while miR-200a-3p is expressed only in pNK cells. For the commonly expressed miRNAs, miR-130b-3p, miR-125a-5p, miR-212-3p, and miR-454 are up-regulated while miR-210-3p and miR-132 are down-regulated in dNK cells compared to pNK cells in all samples.

The differential miRNA expression patterns in dNK and pNK cells might be associated with the acquisition of special phenotypes of NK cell subsets and the modulation of their unique functions. However, investigations into the importance of these mechanisms are still at an early stage. Large follow-up studies are warranted to explore these regulatory networks.

## miRNA regulation of dNK cell immune function during pregnancy

**Fig. 2:**
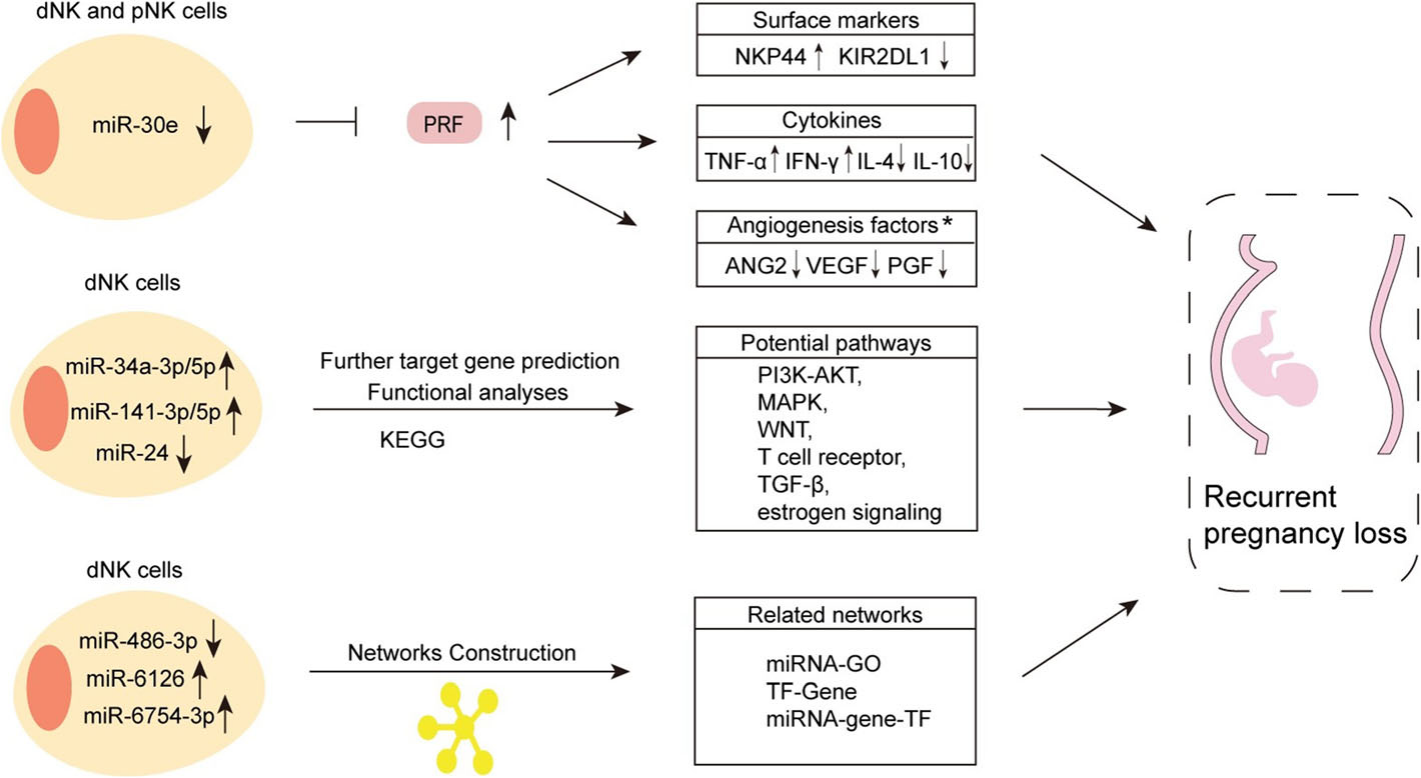
Differentially expressed miRNAs in dNK cells may be associated with RPL. miR-30e, miR-34a-3p/5p, miR-141-3p/5p, miR-24, miR-486-3p, miR-6126 and miR-6754-3p are dysregulated in dNK and pNK cells of RPL patients. These miRNAs mediate their downstream immune-related proteins and regulatory pathways which might be associated with RPL. RPL, Recurrent pregnancy loss; dNK cell, Decidual NK cell; pNK cell, Peripheral NK cell; PRF, Perforin 1. * These immune-related proteins only change in dNK cells

NK cells develop from hematopoietic stem cells and mature after experiencing intermediate states such as NK precursor cells (NKP) and immature NK cells (iNK) [54]. Developing NK cells exhibit a dynamic miRNA expression profile that is substantially different from that of mature NK cells. miRNAs have been highlighted as direct modulators of NK cell fate [55]. However, the underlying, relevant pathways triggering immune responses in pregnancy remain insufficiently understood. Exciting discoveries during the past 10 years have revealed the direct interplay between dysregulated miRNAs in NK cells and gestation outcome (Fig. 2).

### Regulation of dNK cell activation

miR-30e has been implicated in the pathogenesis of a variety of human diseases, such as bladder cancer, breast cancer, and cardiac fibrosis [56–58]. Recently, investigators demonstrated the effects of miR-30e on regulating the functions of pNK and dNK cells at maternal–fetal interface [59]. They found the expression of miR-30e is decreased in both peripheral blood and decidual tissues in RPL patients, suggesting that the alteration of miR-30e may be related to the pathogenesis of RPL. Further results show that miR-30e is down-regulated and its downstream target gene, PRF1 (Perforin 1), is up-regulated in the activated pNK and dNK cells (activated by IFN-α), indicating that miR-30e may be associated with the activation of NK cells by negatively regulating PRF1.

### Regulation of dNK cell cytotoxicity

Furthermore, in pNK and dNK cells, the expression of NKp44, an activating receptor, is reduced and the expression of killer cell immunoglobulin-like receptor, two Ig domains and long cytoplasmic tail 1 (KIR2DL1), an inhibitory receptor, is elevated in the miR-30e mimics group, while NKp44 is increased and KIR2DL1 is inhibited in the miR-30e inhibitors group [59]. NKp44 is responsible for the increase in killing of cell lines and the release of cytotoxic granules [60]. In addition, NKp44 on dNK cells is critical for the tolerance of pregnancies in the uterus [61, 62]. KIR2DL1 is a crucial member of KIR family that could deliver several signals that inhibit cytotoxicity of NK cells [63]. Overall, miR-30e might affect NK cell cytotoxicity by regulating the expressions of NKp44 and KIR2DL1.

### Regulation of dNK cell cytokine secretion

One of main functions of NK cells is the production of cytokines [64]. TNF-α, IFN-γ, IL-4, and IL-10 are spontaneously released by pNK and dNK cells and could be important for maintaining a successful pregnancy [65, 66]. In both pNK and dNK cells, the miR-30e mimics group showed higher mRNA and protein expression of IL-10 and IL-4 compared to the negative control group, and the miR-30e inhibitors group showed lower mRNA and protein expressions of IL-10 and IL-4 compared to the negative control group. The miR-30e mimics group had decreased mRNA and protein expression of TNF-α and IFN-γ, while the miR-30e inhibitors group had elevated mRNA and protein expressions of TNF-α and IFN-γ [59]. These findings suggest that miR-30e in NK cells can enhance the release of IL-4 and IL-10 while repressing the production of TNF-α and IFN-γ.

ANG-2 [67], VEGF [68], and placental growth factor (PGF) [69] have recently been studied for their roles in the development of the placenta and fetus. Women with low VEGF and PGF level are believed to be at higher risk of RPL and SGA [68, 70]. The discovery of miRNAs in dNK cells could potentially affect growth factors production at maternal–fetal interface. For example, in dNK cells, ELISA and qPCR results show that both concentrations and mRNA expression of ANG-2, VEGF, and PGF are increased in the miR-30e mimics group while decreased in the miR-30e inhibitors group [59]. This study illustrates mechanisms by which decreased miR-30e expression in dNK cells may directly affect their functions of promoting fetal development. Specific targeting of miR-30e in dNK cells might contribute to the development of novel intervention strategies (therapies that use miRNA mimetics and anti-miRNA oligonucleotides), aiming at regulating dNK cell induction in RPL and SGA.

### Involvements of functional pathways

A previous study [71] have detected the expression levels of miR-34a, miR-24, miR-155, miR-125a, miR-125b, and miR-141 in dNK cells derived from RPL patients and control groups via qPCR. The results showed that miR-24 is down-regulated while other 5 miRNAs are up-regulated in the RPL group suggesting that these miRNAs may play indispensable roles at the maternal–fetal interface. Furthermore, miR-34a, miR-141, and miR-24 are subjected to target gene prediction and pathway analysis. KEGG analysis of the predicted targets of these 3 miRNAs reveal 140 pathways, including PI3K-AKT, MAPK, WNT, T-cell receptor, TGF-β, and estrogen signaling pathways. These canonical pathways have been identified to affect the development and function of NK cells during pregnancy. For example, activated PI3K-AKT signaling pathway in dNK cells can increase the GzmB, Perforin, IFN-γ, and IL-10 production [72], uncovering miRNA in NK cells may mediate immune mechanisms in gestation. Further research has been conducted with miRNA microarrays to explore miRNA expression profiling in dNK cells derived from RPL patients and healthy controls [73]. The researchers have identified 50 differentially expressed miRNAs, including 49 up-regulated and 1 down‐regulated miRNA in the RPL group (For example, miR-486-3p, miR-6126, and miR-6754-3p). The only down‐regulated one is miR‐486‐3p which has been proven to display dysregulated expression in PE patients compared with the control group [74]. The findings have also exhibited miRNA‐GO, TF‐Gene, and miRNA‐gene‐TF networks that may be associated with regulatory mechanisms of RPL. Importantly, miR-3620‐5p is present in all three networks above, indicating it might be critical in regulating dNK cell function at the maternal–fetal interface.

## Future directions

Currently, a clinical need exists for predictive biomarkers of pregnancy complications, such as RPL [75]. The number of NK cells has already become a clinical reference index for RPL [76], and several circulating miRNAs are also known to be differentially expressed in RPL patients when compared with healthy individuals, acting as potential biomarker candidates [77, 78]. But whether differentially expressed miRNAs in NK cells can be able to more accurately discern normal individuals from those with pregnancy related disorders should be revealed in more in-depth studies. In addition, whether these dysregulated miRNAs in NK cells identify pregnant women who are at risk of pregnancy complications has substantial value for alleviating the impact of gestational diseases. On the other hand, immunosuppressant interventions are commonly used to treat RPL in recent years [79, 80]. It is still unclear that whether the levels of miRNAs in dNK cells could be affected by clinical interventions, which can monitor the effects of interventions on pregnancy complications. For in vivo applications, the potential for miRNA-targeted therapeutics has been indicated [81, 82], and safe miRNA-targeted drugs are attracting much attention from researchers and hold great promise for clinical treatments of pregnancy related diseases.

Lastly and importantly, tre existing literature on dNK cell molecule regulation is very limited; most report on only the expression levels of miRNAs [53, 59, 83], and few are in-depth reports focused on the basic mechanisms of miRNA function. This lack may be related to the difficulty in purifying enough primary dNK cells to meet the requirements of deep sequencing. It is also challenging to conduct gene knockout experiments in primary dNK cells [38]. Additionally, many small molecules including circular RNAs and methylation modifiers (such as N6-methyladenosine and 5-hydroxymethylcytosine) have not been examined in dNK cells. Whether the RNA modifications [84, 85] in dNK cells and pNK cells regulate biological immune functions during pregnancy is worth exploring in the future.

There are functional miRNAs in ESCs [86] (such as miR-149 [87], miR-181a [88], miR-21 [89], and miR-542-3p [90]), influencing the endometrial decidualization process. Regulatory miRNAs in trophoblast cells also contribute to their proliferation, migration, and invasion (such as miR-210 [91], miR-126-3p [92], and miR-23a [93]) during pregnancy. Of note, exosomes play crucial roles in the mediation of cell-to-cell communication at maternal-fetal interface [94, 95], and it is worth exploring that whether above functional miRNAs can be transferred into dNK cells by ESCs/trophoblasts-derived exosomes in pregnancy related disorders, inducing dysfunctions of dNK cells and impairing immune system balance of pregnant women. In turn, NK cell-derived exosomes are identified to mediate cytotoxicity against tumor cells [96, 97], and it is not known whether dNK cell can secrete miRNA-containing exosomes or micro-vesicles into maternal-fetal interface, which can affect systemic inflammation, decidualization and biological behavior of other decidual cells.

## Concluding remarks

**Fig. 3:**
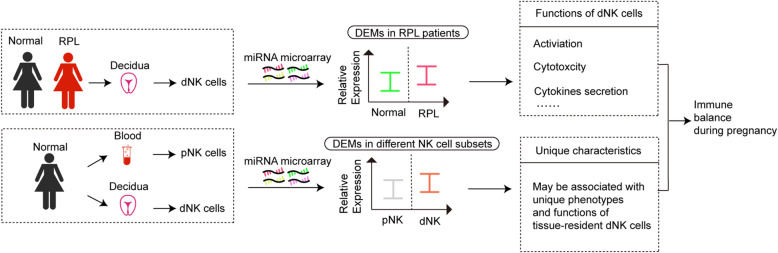
miRNA-mediated dNK cell immune function influences the outcome of pregnancy. miRNAs are present in purified dNK and pNK cells of pregnant women. On the one hand, dysregulated miRNAs in dNK cells might be involved in immunopathological mechanisms of pregnancy related disorders, such as RPL. On the other hand, there are also several differentially expressed miRNAs between dNK and their counterpart pNK cells, which may be also correlated with special characteristics and functions of tissue-resident dNK cells at maternal-fetal interface. dNK cell, Decidual NK cell; pNK cell, Peripheral NK cell; RPL, Recurrent pregnancy loss; DEMs, Differentially expressed miRNAs

We have highlighted the contributions of miRNAs in regulating the development and maturation of NK cells and also controlling the activation of NK cells and their subsequent actions such as generating pro- or anti-inflammatory factors during pregnancy. The dysregulations of miRNAs in both dNK cells and pNK cells could be involved in the immune regulations of pregnancy complications. Therefore, the NK cell-specific targeting of miRNA may present a novel strategy for the prevention and treatment of RPL. In addition, dNK cells exhibit a different miRNA expression pattern compared with their matched pNK cells during the first trimester of pregnancy. Notably, there are also differentially expressed miRNAs in pNK cells at different stages of pregnancy. We hypothesize that these differences may be related to the different numbers or unique functions of NK cells at each pregnancy stage, as well as certain resident characteristics of dNK cells. A more comprehensive understanding of the regulatory mechanisms of miRNAs in dNK and pNK cells and their functional specificities could set a new stage for balancing immune function and damage at the maternal–fetal interface (Fig. 3).

## Data Availability

Literature research results are available from the authors upon reasonable request.

## Abbreviations

AGA: Appropriate-for-gestational-age
ANG: Angiopoietin
COVID-19: Coronavirus disease 2019
CD: Cluster of differentiation
CXCR: C-X-C motif chemokine receptor
DEMs: Differentially Expressed miRNAs
dNK cell: Decidual NK cell
DSCs: Decidual stromal cells
ESCs: Endometrial stromal cells
EVT: Extra-villous trophoblast
FGR: Fetal growth retardation
GNLY: Granulysin
HCMV: Human cytomegalovirus
HGF: Hepatocyte growth factor
IL: Interleukin
IP: Interferon gamma-inducible protein
IFN: Interferon
miRNA: MicroRNA
NCR: Natural cytotoxicity receptor
PE: Pre-eclampsia
PGF: Placental growth factor
pNK cell: peripheral NK cell
PTHLH: Parathyroid hormone-like hormone
RPL: Recurrent pregnancy loss
SGA: Small-for-gestational-age
TGF: Transforming growth factor
TNF: Tumor necrosis factor
Treg cell: Regulatory T cell
uNK: Uterine NK
VEGF: Vascular endothelial growth factor
VSMC: Vascular smooth muscle cell
